# Effects of disbudding on behavior and heart rate during jugular venipuncture in dairy calves

**DOI:** 10.3168/jdsc.2024-0655

**Published:** 2024-11-26

**Authors:** Alycia M. Drwencke, Haley Garcia, Sarah J.J. Adcock, Cassandra B. Tucker

**Affiliations:** 1 Center for Animal Welfare, Department of Animal Science, University of California, Davis, Davis, CA 95616; 2 Animal Behavior Graduate Group, University of California, Davis, Davis, CA 95616; 3 Department of Animal and Dairy Sciences, University of Wisconsin–Madison, Madison, WI 53706

## Abstract

Disbudding is a common painful procedure that alters physiological and behavioral responses in calves. Other routine management procedures are commonly performed on calves while these disbudding wounds are healing, such as vaccine injections, jugular venipuncture to check for passive immunity, and ear tagging. Although disbudding is known to cause long-lasting pain, the effects of an additional invasive procedure on calf behavior and heart rate when wounds are present are understudied. To evaluate the effects of multiple, consecutive routine management procedures, we tested whether calves were more reactive to handling and a jugular venipuncture that occurred 3 d after caustic paste disbudding compared with those that were not disbudded. We assigned 26 heifers to 2 treatments at 3 d of age: sham handling (n = 13) or caustic paste disbudding (n = 13). Three days after paste was applied, heart rate and 3 behaviors (hindleg lifts, foreleg lifts, struggling) were observed while the calves were restrained and a jugular venipuncture was performed. Disbudded calves performed more hindleg lifts during the venipuncture than non-disbudded calves (mean ± SE: paste = 7.5 ± 1.1; control = 4.1 ± 0.6 lifts/min), although we found no evidence of a significant difference in average heart rate (mean ± SE: paste = 142 ± 4; control = 148 ± 7 beats/min), foreleg lifts (mean ± SE: paste = 3.3 ± 0.4; control = 2.9 ± 0.5 lifts/min), or struggling (mean ± SE: paste = 0.03 ± 0.01; control = 0.03 ± 0.01 proportion of handling time). However, foreleg lifts and struggling were likely hindered by the restraint used. Similarly, heart rate may have reached a ceiling effect associated with the stress of handling. The difference in hindleg lifts provides preliminary evidence that disbudded calves are more responsive to the combination of handling and jugular venipuncture 3 d later than non-disbudded controls.

## Highlights

• Painful routine management procedures often occur in close temporal proximity.
• Disbudded calves had more hindleg lifts during a jugular venipuncture than controls.
• Ongoing pain from recent procedures may influence the response to additional events.

## Graphical Abstract

**Figure:**
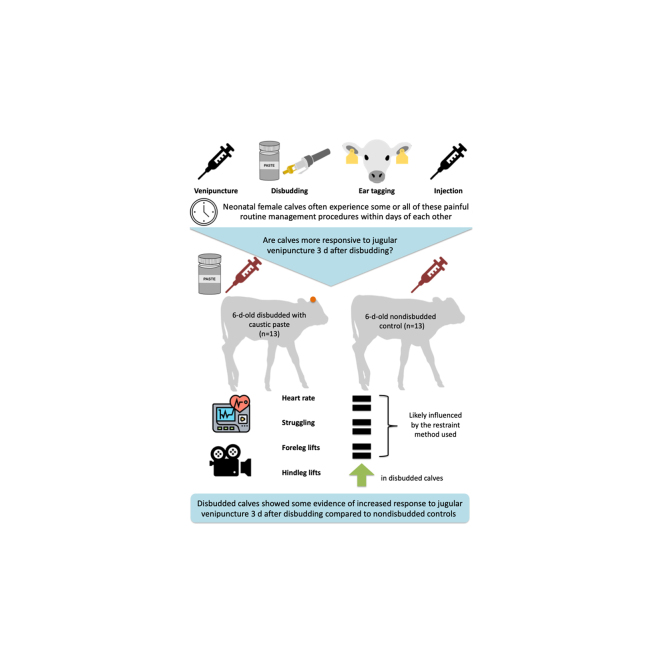
**Summary:** Calves commonly experience multiple painful procedures early in life, such as vaccines, disbudding, supernumerary teat removal, ear tagging, castration (in males), and venipuncture to check for passive immunity. These occur in close temporal proximity, often within days of one another. Although pain from one procedure, such as disbudding, is well documented, we explored calf responses after a second event. Calves that were caustic paste disbudded at 3 days of age showed some evidence of increased responsiveness to jugular venipuncture at 6 days of age compared with non-disbudded controls. These results provide preliminary evidence that disbudding increases responsiveness to additional handling and painful procedures.

Female neonatal dairy calves undergo several routine painful procedures, including disbudding, ear tagging and needle-related processing (e.g., vaccination, venipuncture). These procedures often occur consecutively, with minutes to weeks between each; the order and timing vary from farm to farm. Each procedure has been studied to varying degrees and rarely in combination with others. These procedures alone can affect calf welfare, and some evidence suggests that these effects may be further influenced by previous procedures.

Disbudding is a common painful procedure that prevents horn growth with cauterization from a hot iron (heat) or caustic paste (chemical). Up to 80% and 94% of farms in the European Union and United States, respectively, disbud dairy cattle (Cozzi et al., 2015; USDA, 2018). Disbudded calves have been shown to exhibit pain responses through increased heart rate, cortisol, eye temperature, and behavioral changes such as ear flicks, head shakes, and head rubbing (Stafford and Mellor, 2011). Disbudding creates wounds that persist for an average of 7 to 9 wk from a hot iron or 16 wk from caustic paste and show decreased mechanical nociceptive thresholds during this time (Adcock and Tucker, 2018; Reedman et al., 2022; Drwencke et al., 2023). Behavioral changes in the weeks following the procedure, such as decreased rumination compared with intact controls, can also occur (e.g., Adcock et al., 2023).

Ear tagging occurs on at least 80% of US dairy farms (USDA, 2018) and is required for all cattle in the European Union (European Parliament, 2000). Ear tagging causes damage and pain at the time of the procedure (Lomax et al., 2017) which can lead to worsening, long-lasting wounds (Hayer et al., 2022; Harmon et al., 2023). The long-term effects of ear tagging calves have received little attention, and the timing of ear tagging overlaps with other painful experiences.

Needle-based procedures, including injections (e.g., vaccination) and jugular venipuncture, a method to test for passive immunity (Renaud et al., 2018), can occur in calves while disbudding and ear tag wounds are present. In 2014, 35% of heifer calves were tested for passive immunity in the United States (USDA, 2014). Calf response to needles have been studied in a few contexts. Injecting substances has been found to cause pain in cattle (Ede et al., 2018; Jimenez et al., 2019), but these findings depend on context, including previous experience (Lomb et al., 2021) and the substance injected (e.g., buffered vs. unbuffered lidocaine in cattle: Adcock and Tucker, 2022). Calves will also show escape behaviors during cornual nerve injections (Jimenez et al., 2019). Venipuncture has been shown to induce pain-related behavioral responses in dogs, rabbits, and rats (Flecknell et al., 1990), and dairy calves can exhibit increased cortisol during jugular venipuncture when restrained for 2 min before the needle insertion compared with 0 min (Stilwell et al., 2008). Handling can also be stressful in cattle across contexts (Hemsworth, 2003).

Consecutive painful procedures such as these may also have cumulative effects in young calves. Physiological systems in neonates are often highly plastic and may experience phenotypic alterations during early life experiences such as stress and pain, which can be exacerbated by multiple events (Adcock, 2021). The response to neonatal injury has been modulated by repetitiveness of trauma, injury etiology, age, pain management, and rearing environment (Adcock, 2021). The literature about farm animals contains a few examples where the effects of early insults in combination with routine processing at a later time (e.g., injections) have been studied. Ewes that were tail docked or exposed to a mild infection at 3 to 4 d of age showed more pain-related behaviors than control ewes when giving birth (Clark et al., 2014). Another study found that calf age at the time of disbudding influenced the autonomic nervous system response to an injection at 1 yr of age; although all animals found injections aversive, this experiment lacked a non-disbudded control (Adcock and Tucker, 2020). Last, Osório-Santos et al. (2024) found no evidence that caustic paste disbudding of one horn bud at 10 d of age resulted in altered pain thresholds to hot iron disbudding the other horn bud 30 d later, suggesting the presence of wound tissue is not inherently a risk for a heightened response to a subsequent procedure. Ongoing inflammation from injury has been shown to increase the response to additional stressors in other species (Chapman et al., 2008). Multiple concurrent stressors or painful procedures have been shown to increase animal responsiveness when compared with a single incident (e.g., male dairy calves: Sutherland et al., 2013; beef calves: Lambertz et al., 2015), but there is scant study of consecutive procedures in female dairy calves.

Our objective was to evaluate the effects of an additional routine management procedure on calves when disbudding wounds were present. We predicted that disbudded calves would show a greater response to jugular venipuncture (including more leg lifts and struggling behavior and higher heart rate) than non-disbudded controls. We predicted disbudded calves would be affected by both the stress of handling and pain from the jugular venipuncture. All calves were ear tagged at 2 d of age and given a subcutaneous injection of selenium and vitamin E, making the wounds from ear tags and any subsequent sensitivity to injections part of the known “pain landscape” of our population.

Data were collected from July to October 2021. All procedures were approved by the UC Davis Institutional Animal Care and Use Committee (protocol no. 21601). Thirty-three healthy female calves born between July 3 and October 2, 2021 were assigned in blocks to caustic paste disbudding or non-disbudded control treatments as part of a larger study (Drwencke et al., 2023) using a random number generator without replacement. Treatments were balanced through date of enrollment by breed and birth weight. We used a subset of 26 calves (n = 13/treatment; 10 Holsteins and 3 Jerseys each). The first 6 calves in the larger study were not included as jugular venipuncture training was incomplete, and 1 calf was skipped due to signs of heat stress after disbudding. Calves were used opportunistically for this additional experimental question; the main study sample size was determined using pain sensitivity data as described in Drwencke et al. (2023). At 2 d of age, all calves were ear tagged in both ears (Harmon et al., 2023), given a subcutaneous injection of selenium and vitamin E (BO-SE, Merck Animal Health) and administered 2 mL of a nasal vaccination (Inforce 3, Zoetis). At 3 d of age, disbudded calves were placed in a head restraint (Jimenez et al., 2019) and received a cornual nerve block of 5 mL of 2% unbuffered lidocaine hydrochloride (Vet One) per horn bud, followed by 1 mg/kg of oral meloxicam after release. Horn buds were checked for anesthesia using gentle pinpricks with a clean needle around the bud. Caustic paste (calcium hydroxide 37.8%, sodium hydroxide 24.9%; H. W. Naylor Company Inc.) was rubbed into unshaved horn buds at 0.25 or 0.30 mL per horn bud when calves had a birth weight of <34 kg or ≥34 kg, respectively. Control calves received a sham disbudding at 3 d of age, in which the horn buds were rubbed with a gloved finger and a needleless syringe was touched to the head to mimic the cornual nerve block. All calves were housed individually in outdoor plastic hutches (2.0 × 1.5 × 1.4 m; length × width × height) with an attached wire-fenced pen (2.0 × 1.5 × 0.9 m; length × width × height) below a shade cloth structure. Access to grain (Starter Calf Feed 901033, Associated Feed and Supply Co.) and water was available ad libitum, and milk replacer (26% CP, 16% fat, 15% total solids, mixed as indicated at a rate of 142 g/L of hot water; Calva Products Inc.) was fed twice daily. At the time of the jugular venipuncture, Jerseys and Holsteins were fed 1.4 and 1.9 L of milk, respectively, twice daily (09:00 and 16:00).

A jugular venipuncture was completed by a trained researcher (AMD) after the morning feeding (∼10:00) at 6 d of age, past the 40-h mean terminal half-life of meloxicam in calf blood plasma (Mosher et al., 2012). Before the procedure, the calves were kept in the attached wire-fenced pen and placed in the head restraint to be fitted with a Polar V-800 heart rate monitor (Polar Electro) to collect heart rate (beats/min). A patch of hair (5 × 10 mm) was shaved, and a conductive gel was applied (Spectra 360 gel, Parker Laboratories) under the water-soaked chest strap and monitor. A layer of vet wrap was used around the girth to firmly secure the monitor. Calves were removed from the head restraint and placed in the back-left corner of the pen where the hutch and wire fencing met. The calf was restrained so her shoulders were between the legs of the researcher, and her head was held in place (Figure 1). A jugular venipuncture was performed using a 20-gauge × 38 mm double ended needle, and a maximum of 3 insertions were allowed. Blood was collected into a red-top evacuated tube for serum testing. All venipunctures were video recorded with a Panasonic HC-V550 camera positioned 1 m from the left side of the pen, mounted on a tripod and angled to see the entire calf.

**Figure 1:**
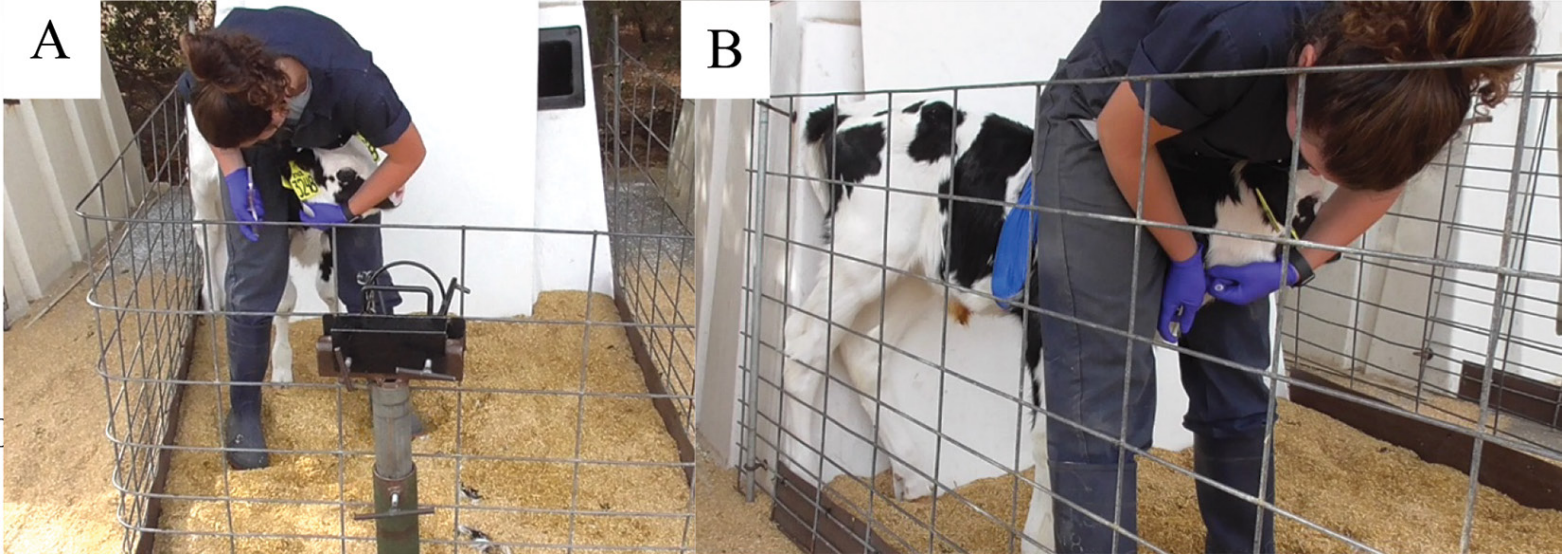
Front (A) and side (B) views of the restraint used during jugular venipuncture at 6 d of age, 3 d after disbudding paste was applied or sham disbudding for control calves. Calves were removed from the head restraint (visible in photo A) and placed in the back-left corner of the pen where the hutch and wire fencing met.

Handling time started when the calf was placed between the researcher's legs and stopped when blood was collected and the calf was released. Videos were continuously observed for the entire handling time in BORIS (Friard and Gamba, 2016) for 3 behaviors. Behaviors included the frequency of hindleg lifts, foreleg lifts, and duration of struggling. Leg lifts were defined as either limb (front or hind) rising away from the ground such that space was visible between the entire hoof and bedding. For both front and hindleg lifts, a new lift was counted each time the hoof came in contact with the bedding. Struggling was classified as the calf pushing forward or pulling away from the human performing the blood draw and restraint, moving their head or body, or both, in the respective direction. A new bout of struggling began when the calf stopped moving their head and body for >1 s. Before data collection began, interobserver reliability was calculated by comparing researchers HG and AMD video coding from 8 calves (intraclass correlation coefficients [**ICC3**] were 0.82, 0.93, and 0.98 for hindleg lifts, foreleg lifts, and struggling, respectively). The BORIS event plots were also inspected for visual agreement between observers. For data collection, all video was watched by 1 person (HG). Intra-observer reliability for HG was determined by watching 7 calves at the start and end of data collection and calculating ICC3 (0.98, 0.97, 0.98 for hindleg lifts, foreleg lifts, and struggling, respectively). Throughout, ICC3 was calculated in R using the “*psych*” package version 2.3.6 (Revelle, 2023). Due to the nature of the disbudding wounds, researchers were not blind to treatment.

All data (Drwencke et al., 2024) and RMarkdown files for analyses and figures, and example procedure video are available in the Dryad repository (see Notes). Statistical analyses were conducted in R version 4.3.1 (R Core Team, 2022). Although we found no difference in handling time between treatments (mean ± SE: control = 163 ± 40 s; paste = 182 ± 36 s; *P* = 0.31) we observed variation among individual calves (47–440 s). To account for differences in the length of handling, struggling was analyzed as a proportion of handling time, leg lifts were converted to the number per minute, and heart rate for each calf was averaged across the handling period. All models were checked for normality and homoscedasticity. Hindleg lifts, foreleg lifts, and heart rate were analyzed using a Welch's 2-sample *t*-test between treatments (t.test function in R). Struggling and handling time data were not normally distributed and were analyzed using a Wilcoxon rank sum test (wilcox.test function in the *stats4* package version 4.3.1).

Table 1 provides the means, SE, and test results for each dependent variable. We found no evidence of a significant difference in heart rate (*P* = 0.45). We also found no evidence of a significant difference in the number of foreleg lifts per minute or proportion of time spent struggling in calves during the venipuncture (*P* ≥ 0.53). Caustic paste–disbudded calves displayed more hindleg lifts during the venipuncture than the non-disbudded calves (*P* = 0.01). A limitation of our work is the lack of a sham jugular venipuncture, making it difficult to disentangle the effects of the needle pain and stress from handling. We expect both stress and pain played a role in our results.

**Table 1:** Mean and SE of the heart rate or the proportion of time that non-disbudded control and caustic paste disbudded calves spent exhibiting each behavior during jugular venipuncture at 6 d of age, 3 d after disbudding paste was applied or sham disbudding procedure

Measure	Control, mean ± SE	Paste, mean ± SE	Test statistic	df	*P*-value	95% CI
Heart rate (beats/min)	149 ± 7	142 ± 4	*t* = −0.77	20.01	0.45	−23.24, 10.77
Hindleg lifts (no./s)	4.1 ± 0.6	7.5 ± 1.1	*t* = 2.72	18.49	0.01	0.76, 5.86
Foreleg lifts (no./s)	2.9 ± 0.5	3.3 ± 0.4	*t* = 0.64	23.45	0.53	−0.89, 1.69
Struggling (duration; proportion of observation period)	0.03 ± 0.01	0.03 ± 0.01	W = 86	N/A^1^	0.96	−0.03, 0.03

1 N/A = not applicable for a metric.

Previous research has shown that disbudding is painful for calves both at the time of the procedure and in the weeks that follow as the wounds heal (e.g., Stafford and Mellor, 2011; Adcock and Tucker, 2018; Casoni et al., 2019). However, little is known about the physiological and behavioral response during a secondary procedure while disbudding wounds are still present, such as a jugular venipuncture. We found some evidence that disbudded calves were more responsive to the combination of handling and venipuncture than non-disbudded controls through an increase in hindleg lifts. We did not find evidence of a difference in foreleg lifts, struggling, or heart rate between treatments.

Both treatments had similarly high heart rates, with averages over 140 beats/min, which is higher than values observed in other studies. Restraint alone or in combination with painful procedures can be stressful for cattle, leading to increased heart rate (e.g., Stewart et al., 2013). Several studies in calves have found heart rate increased in response to restraint and pain, but all had lower values, during baseline and in response, than the current study (e.g., baseline 65 and response 80 beats/min in Stewart et al., 2010; and baseline 85 and response 105 to 115 beats/min in Jimenez et al., 2019). However, physiological responses such as heart rate may have a ceiling (Ede et al., 2019). Although heart rate has been shown to increase in neonatal humans during acute pain (Weissman et al., 2012), the age of our calves could also have influenced the high values we observed. Young calves are reported to have heart rates between 100 and 140 beats/min (Hanson, 2020), with Quevedo et al. (2019) reporting an average of 139 beats/min in 7-d-old calves.

We found no evidence of treatment differences in front leg lifts or struggling and, overall, we saw fewer of these responses than in previous work when expressed on a comparable scale. During a cornual nerve block lasting ∼30 s, calves exhibited an average of 12.0 to 15.4 foreleg lifts/min and 6.0 to 7.8 struggling bouts/min during the injections (Jimenez et al., 2019), whereas we observed an average of 2.9 to 3.3 foreleg lifts/min and scored struggling duration rather than frequency. In other work, 11-m-old heifers struggled 4 times more in a head lock when receiving a vaccine injection versus sham handling (Adcock and Tucker, 2020). However, heifers in both studies had fewer physical limitations; during our venipuncture, calves were restricted in the movement of their shoulders, legs, and head (Figure 1), possibly leading to an inability to exhibit front leg lifts and struggling. Indeed, this restraint put the head and ears in contact with the person performing the venipuncture, and pain associated with the contact between the ears and the person may have also limited their willingness to move the front of their body. We also note that our sample size was smaller than in the previous work (e.g., Adcock and Tucker, 2020), but comparable to others (e.g., Jimenez et al., 2019).

Pain from ear tagging and previous BO-SE injection may have contributed to the affective state and experience of all calves. Ear tagging occurred 4 d before the jugular venipuncture. As part of another study, these calves were monitored for wounds from ear tags (Harmon et al., 2023). Nearly all calves were found to have wounds present at the time of the jugular venipuncture (Harmon et al., 2023). Pain during ear tagging procedures has been evaluated (e.g., Stewart et al., 2013), but persistent pain from long-lasting wounds is possible. Although the acute pain from the BO-SE injection was likely diminished, ongoing aversion from this injection is possible (Ede et al., 2018). The pain landscape may have been increased for both paste-disbudded calves and non-disbudded controls by their ear tag wounds and previous injections, as all calves experienced these procedures.

However, disbudded calves showed some evidence of a heightened response to venipuncture compared with non-disbudded controls. Although hindleg lifts were relatively rare (4.1 vs. 7.5/min), increase of nearly 2 times observed in our study is consistent with other work. Hindleg side steps increased during cornual nerve blocks in calves, reaching 5.6/min when injected with lidocaine and 9.8/min with buffered lidocaine, whereas sham calves averaged 1.6/min during 25 s of observation (Adcock and Tucker, 2022). Hindleg side steps also increased during a more painful cornual nerve block: 7.6 versus 4.6/min, respectively (Jimenez et al., 2019). Hindleg lifts increased to 15.0/min during a 60-s vaccination, compared with 2.0/min during baseline, which was further increased by disbudding at 3 versus 56 d of age (Adcock and Tucker, 2020). The increase in hindleg lifts in the current study provides some evidence that disbudded calves are more responsive than non-disbudded calves to the combination of handling and jugular venipuncture 3 d later.

The underlying mechanism driving increased responsiveness of disbudded calves is not clear with the current experimental design. Ongoing pain from disbudding could be driving the increased response to both handling stress and pain from the venipuncture (Chapman et al., 2008). Systemic changes of the pain pathway could have occurred, but these alterations generally become clearer after the initial wounds have healed (Adcock, 2021). Previous handling experiences from disbudding could also play a role, as negative experience has been shown to increase future response to handling (Goonewardene et al., 1999).

Across the literature, there is mixed evidence showing increased responsiveness to an additional painful procedure. In previously disbudded 1-y-old heifers, the response to an injection differed based on the age at the procedure, though not in a consistent direction (Adcock and Tucker, 2020). In calves with disbudding wounds, and likely ear tag wounds, there was no difference in mechanical stimulation at the site of the injury or a distal location between calves that had 1 horn bud previously disbudded or 1 sham handled (Osório-Santos et al., 2024). Our current work found 1 out of 4 measures differed between disbudded and non-disbudded control calves during a jugular venipuncture. These mixed results highlight the complex nature of studying pain, particularly when combined with a stressful event such as handling, the need for sensitive and specific metrics, and the many factors that can influence an animal's experience.
